# Prevalence and speciation of brucellosis in febrile patients from a pastoralist community of Tanzania

**DOI:** 10.1038/s41598-020-62849-4

**Published:** 2020-04-27

**Authors:** Rebecca F. Bodenham, AbdulHamid S. Lukambagire, Roland T. Ashford, Joram J. Buza, Shama Cash-Goldwasser, John A. Crump, Rudovick R. Kazwala, Venance P. Maro, John McGiven, Nestory Mkenda, Blandina T. Mmbaga, Matthew P. Rubach, Philoteus Sakasaka, Gabriel M. Shirima, Emanuel S. Swai, Kate M. Thomas, Adrian M. Whatmore, Daniel T. Haydon, Jo E. B. Halliday

**Affiliations:** 1 Institute of Biodiversity, Animal Health & Comparative Medicine, College of Medical Veterinary and Life Sciences, University of Glasgow, Glasgow, UK; 2 Sokoine University of Agriculture, Morogoro, Tanzania; 3 OIE/FAO Brucellosis Reference Laboratory, Department of Bacteriology, Animal & Plant Health Agency, Surrey, UK; 4 Nelson Mandela African Institution for Science and Technology, Arusha, Tanzania; 5 Duke Global Health Institute, Duke University, Durham, North Carolina USA; 6 Kilimanjaro Christian Medical Centre, Moshi, Tanzania; 7 Kilimanjaro Clinical Research Institute, Moshi, Tanzania; 8 Centre for International Health, University of Otago, Dunedin, New Zealand; 9 Kilimanjaro Christian Medical University College, Moshi, Tanzania; 10 Division of Infectious Diseases and International Health, Duke University Medical Center, North Carolina, USA; 11 Endulen Hospital, Ngorongoro Conservation Area, Arusha, Tanzania; 12 Programme in Emerging Infectious Diseases, Duke-NUS Medical School, Singapore, Singapore; 13 Directorate of Veterinary Services, Ministry of Livestock and Fisheries, Dodoma, Tanzania

**Keywords:** Bacteriology, Clinical microbiology, Infectious-disease diagnostics, Infectious diseases, Bacterial infection, Risk factors

## Abstract

Brucellosis is an endemic zoonosis in sub-Saharan Africa. Pastoralists are at high risk of infection but data on brucellosis from these communities are scarce. The study objectives were to: estimate the prevalence of human brucellosis, identify the *Brucella* spp. causing illness, describe non-*Brucella* bloodstream infections, and identify risk factors for brucellosis in febrile patients from a pastoralist community of Tanzania. Fourteen (6.1%) of 230 participants enrolled between August 2016 and October 2017 met study criteria for confirmed (febrile illness and culture positivity or ≥four-fold rise in SAT titre) or probable (febrile illness and single SAT titre ≥160) brucellosis. *Brucella* spp. was the most common bloodstream infection, with *B. melitensis* isolated from seven participants and *B. abortus* from one. *Enterococcus* spp., *Escherichia coli*, *Salmonella enterica, Staphylococcus aureus* and *Streptococcus pneumoniae* were also isolated. Risk factors identified for brucellosis included age and herding, with a greater probability of brucellosis in individuals with lower age and who herded cattle, sheep or goats in the previous 12 months. Disease prevention activities targeting young herders have potential to reduce the impacts of human brucellosis in Tanzania. Livestock vaccination strategies for the region should include both *B. melitensis* and *B. abortus*.

## Introduction

Brucellosis is a globally widespread zoonotic disease^1,2^, reported as a top ten zoonosis in terms of impact on human health and economics of impoverished communities^3^ and ranking in the top five diseases causing livestock losses worldwide^4^. The *Brucella* species that most commonly cause human infections are *B. melitensis, B. abortus*, and *B. suis*^1,5^. These species are classically associated with small ruminants, cattle, and swine, respectively but transmission between animal hosts is possible^6,7^. Transmission from animals to people is typically via direct contact with infected animals, foodborne transmission, or indirect contact with contaminated environments^2^. Human to human transmission is negligible^8^. Human brucellosis typically presents as non-distinct acute or chronic febrile illness^9,10^, and is frequently clinically misdiagnosed as other causes of febrile illness, such as malaria or typhoid fever^10,11^. Brucellosis is seldom fatal, but chronic infection is often debilitating and severe complications may occur^10^.

Although not as well recognised as in northern Africa, the Middle East or central Asia, brucellosis is endemic in many regions of sub-Saharan Africa^12,13^. Within pastoral systems where people live in close contact with livestock, there is increased risk of human infection^14^. Approximately 16% of the population of sub-Saharan Africa practice pastoralism. Within Tanzania, it is estimated that up to 40% of the population practices exclusive pastoralism^15^. As is true regionally, data on brucellosis prevalence and incidence are limited for these communities^16,17^.

Hospital-based febrile surveillance studies of a predominantly urban population in Tanzania, have generated prevalence estimates of 3.5% for confirmed brucellosis in 2007–2008^18^ and 8.9% for confirmed or probable brucellosis in 2012–2014^19^ by *Brucella* microagglutination testing. Serological testing cannot be used for *Brucella* spp. identification^20^. *Brucella* spp. can be identified through molecular diagnostic analyses of DNA obtained from culture isolates^21^ or clinical samples, or with phenotypic testing of isolates^2^. Studies in sub-Saharan Africa that have identified the infecting *Brucella* spp. from human infections are rare^6,22^. There are no human *Brucella* spp. isolates from Tanzania recorded to date. Livestock isolates from Tanzania are also scarce, but both *B. melitensis* and *B. abortus* have been isolated, from goats and cattle, respectively^23,24^. Identification of the *Brucella* spp. causing human illness is vital to inform understanding of the most likely animal source population(s) and the design of vaccination strategies, as brucellosis vaccines are animal host specific.

*Brucella* spp. are typically a relatively infrequent cause of human bloodstream infections in sub-Saharan Africa as compared to other regions. In sub-Saharan Africa the predominant causes of bloodstream infections are *Salmonella enterica*, *Streptococcus pneumoniae*, *Staphylococcus aureus*, and *Escherichia coli*^25,26^. Bloodstream infection data from communities considered at a high risk of brucellosis within sub-Saharan Africa are scarce.

There are few studies in East Africa that have identified risk factors for acute brucellosis infection as compared to *Brucella* spp. exposure^19,27–29^. One study, conducted within a largely urban population in Tanzania identified assisting in small ruminant births and contact with cattle as risk factors for human brucellosis^19^. Consumption of boiled or pasteurised dairy products was a protective factor^19^.

To our knowledge, no studies have identified the *Brucella* spp. responsible for human brucellosis in Tanzania. The objectives of this study were to estimate the prevalence of human brucellosis, identify the *Brucella* spp. causing human illness, characterise additional causes of bloodstream infections, and identify risk factors for brucellosis in febrile hospital patients from a pastoralist community of Tanzania.

## Materials and Methods

### Study setting and population

The study was conducted at Endulen Hospital in the Ngorongoro Conservation Area (NCA), northern Tanzania. The NCA is a multiple land use area of 8,292 km^2^, designated for the pastoralist activities of the local Maasai community, the conservation of wildlife, and tourism^30^. Livestock keeping is ubiquitous among the Maasai. Cattle, sheep, and goats are the predominant livestock species kept and are managed extensively in mixed herds^30^. Endulen Hospital is the only hospital within the NCA. It is a 110-bed hospital providing both outpatient and inpatient services to a population of approximately 77,000 persons predominantly resident within the NCA^31^.

### Study eligibility

All individuals seeking care at the outpatient department of Endulen Hospital were eligible for screening, which was integrated into the patient triage processes and performed by Endulen Hospital staff. All individuals aged ≥two years with reported fever within the past 72 hours and/or a tympanic temperature of ≥38.0 °C at presentation were eligible for enrolment. After initiation of the outpatient visit and routine clinical assessment by Endulen Hospital staff, eligible patients were approached by a member of the study team for informed consent to participate in the study.

### Sample collection, malaria testing and questionnaire data collection

After cleaning the participant’s skin with isopropyl alcohol and povidone iodine, blood was drawn from study participants for culture, *Brucella* serology, and malaria testing. The target blood volume at enrolment was 40 mL, enabling distribution of two 10 mL volumes into BacT/ALERT (BioMérieux, Durham, NC, USA) aerobic blood culture bottles for automated culture, 10 mL into Castañeda media^32^ (media prepared at the Animal and Plant Health Agency (APHA), Weybridge, UK), and 10 mL into a plain vacutainer (BD, Franklin Lakes, NJ, USA) for serology. For study participants weighing less than 25 kg, target blood volumes were determined based on weight^33^ and paediatric BacT/ALERT bottles were used for automated culture. For all study participants, a fill order algorithm was followed, with sample collection prioritised as follows: first automated aerobic blood culture bottle, plain vacutainer, Castañeda culture bottle, and second automated aerobic blood culture bottle. Malaria rapid diagnostic testing was performed directly from the sample collection syringe using the SD BIOLINE Malaria Ag P.f/Pan rapid diagnostic test (Standard Diagnostics/Abbott, Abbott Park, IL, USA) or *CareStart* Malaria HRP2 (Pf) (ACCESS BIO, INC. Somerset, NJ, USA).

A member of the study project team administered a structured, closed-ended questionnaire for each study participant (Supplementary Methods). Question topics included: demographic data, symptoms of current and recent illness, dietary practices, and animal-related activities. Data collected from study participant clinical records included the the initial clinical diagnosis recorded and any drug treatments prescribed on the day of hospital presentation (i.e., before any study diagnostic results were available).

All participants were approached for convalescent-phase blood sampling at their homes four to six weeks after enrolment. Up to 10 mL blood was collected into a plain vacutainer for convalescent-phase serology.

### Blood culture

Inoculated culture bottles were packed for transportation to achieve a target temperature range of 4–10 °C^34^. Tinytag Transit 2 Temperature Data Loggers (Gemini Data Loggers Ltd, Chichester, UK) were used to monitor transport temperature. Inoculated culture bottles were transferred to the Kilimanjaro Clinical Research Institute (KCRI) in Moshi, Tanzania, for laboratory processing the day after inoculation. Time and date of inoculation and receipt at the laboratory were recorded. Prior to incubation, the bottom of the BacT/ALERT blood culture bottle was visually assessed to confirm no colorimetric change. BacT/ALERT bottles were then loaded into the BacT/ALERT 3D instrument and incubated for up to 5 days. Castañeda bottles were incubated in a CO_2_ incubator at 5–10% CO_2_ and 37 °C. Bottles were examined for growth every 72 hours for up to 35 days. For two periods during the study (first from 30 April 2017 to 16 June 2017 inclusive and second from 13 July 2017 to 30 September 2017 inclusive) BacT/ALERT bottles were also processed by manual culture methods due to technical malfunction with the BacT/ALERT system. Standard methods were used for identifying isolates^35,36^. Isolates of gram-negative *coccobacilli* that had positive reactions for urease, catalase and oxidase were classified as presumptive *Brucella* and stored on Microbank beads (Pro-Lab Diagnostics, Bromborough, UK) at −70 °C. Culture bottles were classified as adequately filled if the blood volume added met the supplier’s recommended volume +/− 20%. The following organisms were considered likely contaminants: non-*anthracis Bacillus* spp., *Corynebacterium* spp., *Escherichia vulneris*, *Micrococcus* spp., coagulase-negative *Staphylococcus* spp., viridans streptococci. Additionally, *Pantoea* spp. was identified in three bottles of Castañeda media and was also classified as a likely contaminant in our analysis. The results of positive blood culture were shared immediately with the clinical team at Endulen Hospital to inform clinical management.

### *Brucella* speciation

Presumptive *Brucella* spp. isolates were shipped on dry-ice to APHA, UK, for confirmatory testing. Identification of *Brucella* species by culture was performed according to an established typing scheme^37^. Crude lysates for use as a template for PCR were prepared by suspending a single colony from solid media in 500 µL of nuclease-free water and heating (100 °C for 10 minutes). Molecular confirmation of culture-based species typing was performed using multiplex conventional PCR^38^, quantitative PCR^39^ and application of a nine locus *Brucella* spp. multilocus sequence typing scheme^40,41^.

### *Brucella* serology

Filled vacutainer tubes were inverted five times immediately after blood collection and kept at ambient temperature for 45–60 minutes to ensure clotting prior to centrifugation at 1300–1500 g for 10 minutes. Serum was pipetted into cryovials and stored temporarily at 4 °C at Endulen Hospital before being transported at 4–10 °C to KCRI. Tinytag Transit 2 Temperature Data Loggers monitored transport temperature of serum samples. Sera were stored at −80 °C on arrival at KCRI. Sera were shipped on dry ice to the APHA, for serological testing using the Serum Agglutination Test (SAT). Standardised antigen for the detection of antibodies to *B. abortus, B. melitensis* and *B. suis* was used (RAA0054, APHA, Weybridge, UK) at a working strength to give 50% agglutination with a 1/650 dilution of the International Standard Serum to *B. abortus*. Samples were screened in a microtitre plate at serum dilutions of 1/5, 1/10, 1/20 and 1/40, with total volume per well of 200 µl. Any sample showing agglutination in any screening titre was then retested using the tube test format to confirm the final titre.

### Brucellosis case definition

Brucellosis cases were defined as meeting the study criteria for febrile illness of reported fever within the past 72 hours, and/or a tympanic temperature of ≥38.0 °C at hospital presentation, plus laboratory evidence of infection. Laboratory evidence of confirmed brucellosis was defined as a blood culture positive for *Brucella* spp. or a four-fold or greater rise in *Brucella* antibody titre between acute and convalescent serum samples. Laboratory evidence of probable brucellosis was defined as a SAT antibody titre of ≥160 in either acute or convalescent-phase serum^42^.

### Data management and statistical analyses

A sample size calculation was performed to estimate the number of enrolled febrile hospital participants required in order to (i) detect a minimum number of blood culture positive individuals and (ii) estimate brucellosis case prevalence (based on culture and serologically identified cases). Assuming that 3% of presenting febrile individuals would be *Brucella* blood culture positive as in a previous study in Egypt^43^, it was estimated that a sample of 348 individuals was needed to detect a minimum of six *Brucella* blood culture positive cases with 95% probability. Based on an assumed prevalence of 9%^18^, it was estimated that a sample of 126 individuals would be required to estimate brucellosis case prevalence with precision of 5% and 80% power.

Data were entered using the OpenText TeleForm system (OpenText, Waterloo, Ontario, Canada) into an Access database (Microsoft Corporation, Redmond, WA, USA). Data manipulation and statistical analyses were performed using R^44^. Proportions were reported within exact binomial confidence intervals. Generalised linear models were used to evaluate associations between brucellosis case status (positive or negative, combining confirmed and probable cases) and clinical data. Univariable logistic regression models were used to investigate pairwise associations between brucellosis case status and candidate risk factors for human brucellosis. Questionnaire variables were selected for evaluation based on the previously identified or biologically plausible risk factors. The following variables were evaluated: age (years), sex, and a series of livestock-related risk variables occurring within the previous 12 months. In each case, livestock refers to cattle, sheep and/or goats. Variables evaluated were: assisting livestock parturition, occurrence of livestock abortion or still-born offspring in flock/herd, milking livestock, herding livestock, contact with livestock waste (e.g., cleaning animal enclosures or in the construction of buildings), slaughtering or butchering livestock, consumption of raw meat (including offal and raw animal blood) from cattle, sheep and/or goats, and consumption of raw dairy products from cattle, sheep and/or goats. Univariable models were evaluated using likelihood ratio tests (LRT), with a significant p value ≤0.05. Given the small number of positive brucellosis cases and high potential for data overfitting, variable selection was performed using lasso regression using the R package glmnet^45^. Variables retained in the lasso regression were fitted in a multivariable logistic regression model and evaluated using LRT. Odds ratios (OR), adjusted odds ratios (aOR), and 95% confidence intervals (CI) were estimated.

### Research clearance and ethics

Approval to conduct the the study was granted by the Tanzania Commission for Science and Technology, Tanzania Wildlife Research Institute and the Ngorongoro Conservation Area Authority. Ethical approval was granted by the Kilimanjaro Christian Medical Centre (KCMC) Ethics Committee (698); National Institute of Medical Research (NIMR), Tanzania (NIMR/HQ/R.8c/Vol. I/1140) University of Otago Human Ethics Committee (H17/052), and University of Glasgow College of Medical, Veterinary and Life Sciences Ethics Committee (200140149). The research was performed in accordance with the guidelines and regulations prescribed by the above organisations. Written informed consent for study participation was obtained from each participant and/or their legal guardian, using forms translated into Swahili and verbal translation into Maa when needed.

## Results

**Figure 1:**
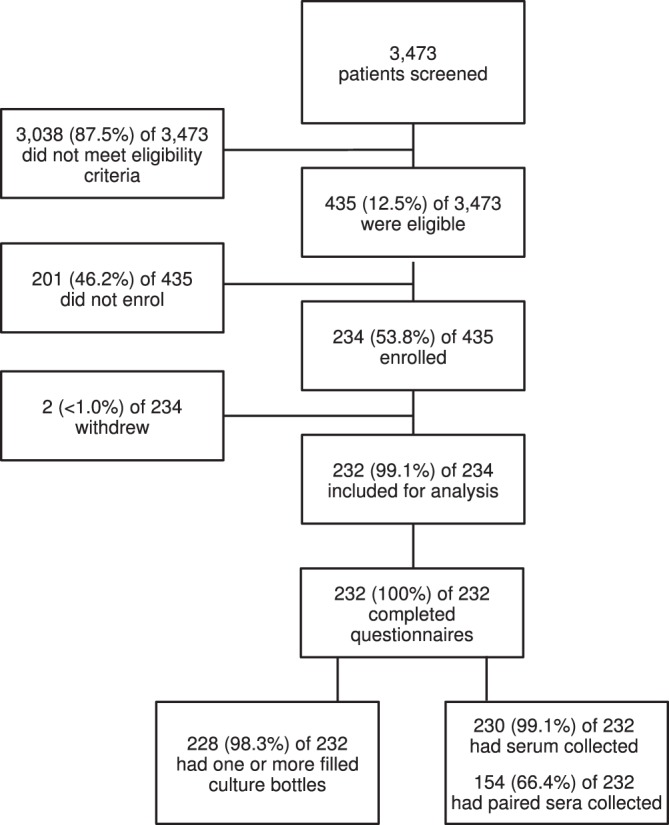
Flowchart describing study participant screening, enrolment and data collection steps, Endulen Hospital, Tanzania, 2016–2017.

From 15 August 2016 to 11 October 2017, 3,473 patients were screened for enrolment into the study. Of the 3,473 screened patients, 435 (12.5%) were eligible for inclusion in the study. A total of 230 participants enrolled and contributed data for the study analyses (Fig. 1).

### Blood culture and *Brucella* speciation

Fourteen (6.1%) of 228 participants with one or more inoculated blood culture bottle had a bloodstream infection. *Brucella* spp. was the most common bloodstream infection. Eight (3.5%) of 228 participants were *Brucella* spp. culture positive. Six other pathogenic bacterial species were identified, each in a single participant (Table 1). *Brucella* spp. isolates from seven (3.0%) participants were identified as *B. melitensis* while *B. abortus* was isolated from one participant (0.4%). The results of molecular speciation assays were congruent with phenotyping. Multilocus sequence typing (MLST) identified all seven *B. melitensis* isolates as sequence type (ST) 12, while the single *B. abortus* isolate was identified as ST32.

**Table 1:** Bacterial pathogens recovered from study participants at Endulen Hospital, Tanzania, 2016-2017 (n = 228), ordered by frequency of detection.

Microorganism		Number (%) of participants in which organism was detected
*Brucella* spp.	*B. melitensis*	7 (3.1)
	*B. abortus*	1 (0.4)
*Enterococcus* spp.		1 (0.4)
*Escherichia coli*		1 (0.4)
*Salmonella enterica*		1 (0.4)
*Salmonella enterica* serovar *Typhi*		1 (0.4)
*Staphylococcus aureus*		1 (0.4)
*Streptococcus pneumoniae*		1 (0.4)

In total, 601 culture bottles were inoculated, with one or more bottles inoculated for 228 (98.3%) of 232 participants. Of the 601 culture bottles, 531 (88.4%) bottles collected from 215 participants were adequately filled and 21 (3.5%) of 601 were contaminated. The median time interval between sample collection and processing at the laboratory was 25.9 (interquartile range 24.8 to 27.0) hours. A total of 77 (85.0%) of 91 shipments of inoculated culture bottles stayed within the target temperature range, all inoculated culture bottles were processed in the laboratory. The comparison of *Brucella* spp. isolation success from adequately filled culture bottle types is given in Table 2. *Brucella* spp. was identified by culture from 16 bottles from the eight culture positive participants, twelve of which had adequate fill volumes. *Brucella* spp. was identified in the Castañeda media bottle for all eight of the culture positive individuals. *Brucella* spp. was identified in one or more of the inoculated BacT/ALERT bottles for five of the eight culture positive individuals including adequate and non-adequate fill volumes. The details of *Brucella* spp. isolation success from the individual culture bottles used for the eight *Brucella* spp. positive individuals is given in Supplementary Fig. S1.The combination of BacT/ALERT bottles used for participants varied throughout the timeline of the study, largely governed by stock and supply variation (Table 2).

**Table 2:** Comparative *Brucella* spp. detection from different combinations of culture bottles, including adequately filled bottles only (n = 531), Endulen Hospital, Tanzania, 2016–2017.

Bottle pair (Bottle A vs Bottle B)	Number of bottles in which *Brucella* spp. was detected		
	Bottle A only	Bottle B only	Both bottles
SA1 vs SA2	0	0	1
SA1 vs FAP1	0	0	0
SA1 vs CAS	0	1	3
SA2 vs CAS	0	0	1
FAP1 vs FAP2	0	0	0
FAP1 vs CAS	0	1	0
FAP2 vs CAS	0	0	0
PF1 vs PF2	0	0	0
PF1 vs PFP	0	0	0
PF1 vs CAS	0	0	0
PF2 vs CAS	0	0	0
PFP vs CAS	0	1	1

Bottle type abbreviations: SA - standard aerobic media; FA - fastidious antimicrobial neutralisation media; FAP - fastidious antimicrobial neutralisation plus media; CAS - Castañeda media; PF - paediatric fastidious antimicrobial neutralisation media; PFP - paediatric fastidious antimicrobial neutralisation plus media; 1 and 2 numbering indicates the order of bottle inoculation when two identical bottle types were filled. For participants ≥25 kg, samples were initially inoculated into two SA bottles, which was later adjusted to one SA and one FAP bottle. During periods with no supply of SA bottles, two FAP bottles were inoculated. For participants <25 kg, samples were initially inoculated into two PF bottles, which was later adjusted to one PF and one PFP bottle.

### *Brucella* serology

Of 232 participants, 230 had acute and/or convalescent serum collected. Paired acute and convalescent phase samples were collected for 154 (70.0%) of 230 participants: 70 participants had acute serum collected only; and six had convalescent serum only, due to an insufficient blood volume collected at acute hospital presentation. Eleven (4.8%) of 230 participants met the case definition for probable brucellosis with one or more SAT antibody titre ≥160. One additional participant met the case definition for confirmed brucellosis based on serology data alone, showing a four-fold rise in antibody titre.

### Brucellosis case classification

A total of nine (3.9%) of 230 participants met criteria for confirmed brucellosis; eight based on blood culture and one by seroconversion. Six (75.0%) of the eight blood culture positive participants had an acute phase SAT titre ≥160, with four of the six also having a convalescent phase SAT titre ≥160. In total, 14 (6.1%) of 230 participants, met the study definition for a probable or confirmed brucellosis case. The correspondence between culture and serology results for study participants and details of the dates of culture positives are shown in Supplementary Fig. S2.

### Participant characteristics, clinical presentation and management

**Figure 2:**
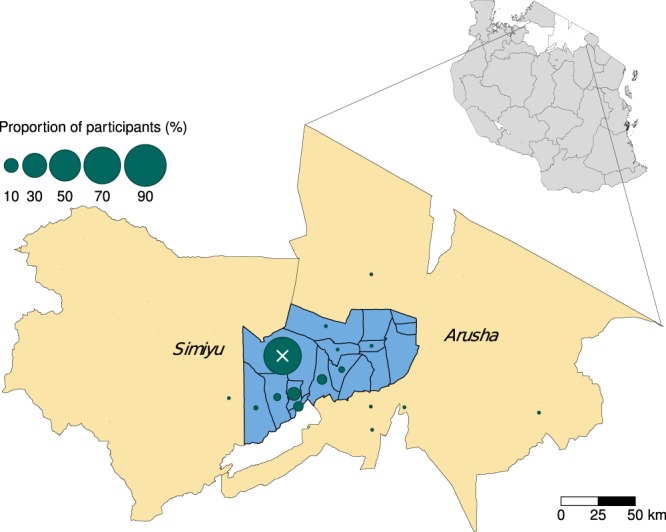
Map showing the position of the Ngorongoro Conservation Area (NCA) (blue shading) within Arusha region and adjacent to Simiyu region (beige shading), Tanzania. Polygon boundaries are shown for all villages within the NCA (blue shading). Green circles show the proportion of enrolled study participants from different villages. The white X indicates the location of Endulen Hospital within Endulen village. In the top right insert, white polygons show Arusha and Simiyu region locations within an outline map of Tanzania (grey shading). Map created using R software version 3.6.1^44^ and the tmap R package^60^. Shapefiles for administrative boundaries from the 2012 census were sourced from the Tanzania National Bureau of Statistics.

Study participants had a median (range) age of 27 (2, 78) years and brucellosis cases had a median (range) age of 11 (7, 20) years. Ten (71.4%) of 14 brucellosis cases were male and 13 (92.9%) reported their tribe as Maasai. Of 232 study participants, 226 (97.4%) reported residence in the NCA with 170 (73.3%) from Endulen village. Residence was also reported in the larger Arusha region and adjacent Simiyu region (Fig. 2). All brucellosis cases were participants living within the NCA.

The relationship between clinical symptoms and brucellosis case status, as well as details of participant clinical management are shown in Table 3. Brucellosis cases were significantly more likely to report night sweats and less likely to report back pain as compared to non-cases (Table 3). Brucellosis case status was significantly associated with receiving a presumptive diagnosis of brucellosis at hospital presentation (LRT *χ*_2_ = 4.82, df = 1, p = 0.03, n = 188) and admission to the inpatient ward of the hospital (LRT *χ*_2_ = 4.97, df = 1, p = 0.03, n = 191). Sixteen (7.0%) of 230 participants, were prescribed a drug regimen consistent with treatment for brucellosis at their initial visit. All of the participants treated for brucellosis had a presumptive diagnosis of brucellosis and treatment for brucellosis was significantly associated with case status as defined by this study (LRT *χ*_2_ = 6.71, df = 1, p = <0.01, n = 230). Data on treatment regimens prescribed after the provision of blood culture results (e.g., after the date of the initial visit) are not included in our dataset. Six (2.6%) of 230 participants had a test positive for malaria. No brucellosis cases were malaria positive.

**Table 3:** Clinical symptoms of study participants based on participant report of symptoms experienced during current illness, Endulen Hospital, Tanzania, 2016–2017.

		Case population n/N (%)	Non-case population n/N (%)	OR (95% CI)	OR p value
**Symptom**					
Reported fever in past 2 weeks		13/14 (92.9)	212/215 (98.6)	0.18 (0.02–3.86)	0.16
Fever type	Continuous	0/13 (0.0)	9/211 (0.04)	Ref	—
	Intermittent	13/13 (100)	202/211 (95.8)	Inf (-Inf – Inf)	0.99
Unit of fever duration	Days	11/13 (84.6)	192/211 (91.0)	Ref	—
	Months	2/13 (15.4)	16/211 (7.58)	2.18 (0.32–9.1)	0.34
	Years	0/13 (0.0)	3/211 (1.42)	0.00 (-Inf – Inf)	0.99
Night sweats		13/14 (92.9)	140/210 (66.0)	6.50 (1.26–119.3)	0.07
Fatigue		11/14 (78.6)	192/213 (90.1)	0.40 (0.11–1.87)	0.19
Joint pain		12/14 (85.7)	190/210 (90.5)	0.63 (0.16–4.24)	0.56
Swollen joints		3/14 (21.4)	30/209 (14.4)	1.63 (0.36–5.58)	0.47
Myalgia		5/13 (38.5)	83/201 (41.3)	0.89 (0.26–2.76)	0.84
Back pain		6/14 (42.9)	152 /213 (71.4)	0.30 (0.10–0.90)	0.03
Headache		12/14 (85.7)	185/214 (86.4)	0.93 (0.24–6.24)	0.93
Anorexia		7/14 (50.0)	105/215 (48.8)	1.05 (0.35–3.16)	0.93
**Clinical Management**					
Presumptive diagnosis including brucellosis		6/13 (46.2)	32/175 (18.3)	3.83 (1.16–12.30)	0.02
Admission to hospital		3/14 (21.4)	7/177 (4.0)	6.62 (1.30–27.75)	0.01
Initial treatment for brucellosis		4/14 (28.6)	12/216 (5.6)	6.80 (1.68–23.91)	<0.01

Reported % reflects the denominator appropriate for each symptom. Univariable analysis odds ratio (OR), 95% confidence intervals (CI) and p values are given. OR, CI and p values reported to two decimal places.

### Risk factors for brucellosis

The univariable associations between the selected risk variables and brucellosis case status are shown in Table 4. Risk factor variables significantly associated with brucellosis in febrile hospital participants in univariable models were: age of participant (LRT *χ*_2_ = 20.05, df = 1, p = <0.01, n = 229), with probability of brucellosis declining with increasing age in years; sex (LRT *χ*_2_ = 4.76, df = 1, p = 0.03, n = 230), with a greater probability of brucellosis in male participants; and herding livestock in the last 12 months (LRT *χ*_2_ = 14.09, df = 1, p = <0.01, n = 219), with participating in herding cattle, sheep and/or goats associated with increased probability of brucellosis. The lasso regression indicated that herding livestock and age variables should be retained. Variables significantly associated with brucellosis in febrile hospital participants in the final multivariable model were: age of participant (LRT *χ*_2_ = 18.17, df = 1, p = <0.01, n = 219), with probability of brucellosis declining with increasing age in years; and herding livestock in the last 12 months (LRT *χ*_2_ = 11.71, df = 1, p = <0.01, n = 219), with participating in herding cattle, sheep and/or goats associated with increased probability of brucellosis (Table 4).

**Table 4:** Univariable and multivariable risk factor analyses for brucellosis case status among febrile hospital participants, Endulen Hospital, Tanzania, 2016–2017.

Variable		Case population n/N (%)	Non-case population n/N (%)	Univariable	Univariable	Multivariable	Multivariable
				OR (95% CI)	OR p value	aOR (95% CI)	aOR p value
Age (years)				0.89 (0.83, 0.95)	<0.01	0.88 (0.81, 0.94)	<0.01
Sex	Female	4/14 (28.6)	126/216 (58.3)	Ref	—		
	Male	10/14 (71.4)	90/216 (41.7)	3.50 (1.13, 13.08)	0.04		
Assisted in livestock parturition		3/14 (21.4)	54/213 (25.4)	0.80 (0.18, 2.69)	0.74		
Livestock abortion or still-born offspring in herd/flock		8/12 (66.7)	80/203 (65.0)	3.08 (0.94, 11.83)	0.07		
Milked livestock		8/14 (57.1)	75/211 (35.5)	2.42 (0.81, 7.59)	0.11		
Herded livestock		12/14 (85.7)	73/205 (35.6)	10.85 (2.86, 70.95)	<0.01	10.16 (2.49, 69.75)	<0.01
Contact with livestock waste		7/14 (50.0)	112/214 (52.3)	0.91 (0.30, 2.75)	0.87		
Slaughtered or butchered livestock		9/14 (64.3)	175/213 (82.2)	0.39 (0.13, 1.33)	0.11		
Consumed raw meat, offal or animal blood		4/14 (28.6)	56/215 (26.0)	1.14 (0.30, 3.55)	0.84		
Consumed raw dairy products		7/14 (50.0)	55/215 (26.0)	2.91 (0.96, 8.86)	0.06		

Odds ratios (OR), adjusted odds ratios (aOR), 95% confidence intervals (CI) and p values are shown. OR, aOR, CI and p values reported to two decimal places. The term livestock refers to cattle, sheep and/or goats. Consumption practices also refer to products from cattle, sheep or goats. The period of reference for all livestock variables was the previous 12 months.

## Discussion

We found that 3.9% of febrile participants had confirmed brucellosis and 6.1% met critieria for confirmed or probable brucellosis in a pastoralist area of Tanzania. *Brucella* spp. was the most commonly identified bloodstream infection in this population and both *B. melitensis* and *B. abortus* were isolated, with a predominance of *B. melitensis*. Risk factor analysis indicated that young livestock herders were at particular risk of infection.

To our knowledge, this is the first study to characterise bloodstream infections in a pastoralist community of Tanzania. *Brucella* spp. was the most commonly identified bloodstream infection in this population, and this contrasts with previous studies which have typically identified *Brucella* spp. as a relatively rare bloodstream infection in sub-Saharan Africa^25^. Several more commonly reported bloodstream infections, including non-typhoidal serovars of *Salmonella enterica*, *Staphylococcus aureus, Streptococcus pneumoniae*, and *Escherichia coli* were also identified in this study population, but at lower relative frequency as compared to *Brucella* spp.^25,26^. Malaria was less prevalent than brucellosis in our study participants, as has been reported previously for northern Tanzania^46^.

Previous examples of isolation and identification of *Brucella* spp. in humans within sub-Saharan Africa are rare^6^. Our study identified *B. melitensis* as the predominant cause of brucellosis, and *B. abortus* was also identified in one participant, confirming the role of both *Brucella* species as causes of human illness in this setting. The *Brucella* genotypes identified represent sequence types that are almost exclusively associated with Africa, but widespread within the continent. *B. melitensis* ST12 has been identified previously in human cases associated with Somalia, Ethiopia, Eritrea, Kenya, Uganda, Nigeria, and Egypt, and in animal infections from Zimbabwe, South Africa, and a single Tanzanian livestock isolate^47^. Fewer isolates of *B. abortus* ST32 than *B. melitensis* ST12 have been reported from livestock and humans, in Kenya, Chad, and Rwanda^47^.

The isolation of both *B. melitensis* and *B. abortus* indicates that the host species for brucellosis in northern Tanzania are likely to include several of the key livestock species kept in this study area, such as cattle, sheep, and goats. Camels are not common within the study area. Consistent with the predominance of *B. melitensis* in this study, previous work in the region has identified small ruminants specifically as the most likely sources of human *Brucella* exposure^48,49^. The NCA is a wildlife conservation area and common species including buffalo (*Syncerus caffer*) and wildebeest (*Connachaetes taurinus*) are known hosts of *Brucella* spp.^50^. Transmission from livestock to humans is likely to account for the majority of human infections, but transmission from wildlife to humans in this setting cannot be ruled out.

The overall prevalence of brucellosis identified in this study is consistent with the findings of previous febrile surveillance studies conducted in Moshi, Tanzania, providing additional evidence of the health impacts of brucellosis in northern Tanzania. Using similar diagnostic approaches these previous studies have detected serologically confirmed brucellosis at between 3.5% and 6.9%^18,19^. All confirmed cases in these previous studies were defined by seroconversions^18,19^. In this present study, the majority of confirmed cases were blood culture positive and the number and proportion of confirmed cases defined by seroconversion was lower than expected. This difference in the ratio of culture to seroconversion-defined confirmed cases may relate to the timing of participant presentation at hospital. *Brucella* spp. isolation is typically more likely early in acute infection^21^. Confirmation of cases based on demonstration of seroconversion is most likely when an acute phase sample is collected very soon after initial infection and a convalescent phase sample is also collected several weeks after initial infection. Interestingly, the majority of blood culture-confirmed brucellosis cases identified in this study also had acute phase sample SAT titre ≥160, indicating that they had already seroconverted by the time of presentation and also had bacteraemia that was still detectable by blood culture (Supplementary Fig. S2). The majority of participants in this study lived close to Endulen Hospital, whereas earlier studies in Moshi were conducted at a referral hospital^18,19^. All confirmed cases in the previous studies were defined by seroconversion and it is plausible that longer time to presentation explain the absence of culture-confirmed cases in these studies^18,19^. Two *Brucella* spp. isolates have been obtained from similar ongoing studies in Moshi (Crump JA & Rubach MP, unpublished data). Patients attending an urban referral hospital may have had increased access to antimicrobials prior to hospital presentation, again reducing chances of positive culture. Additionally, our study was designed to preferentially detect acute brucellosis in acutely febrile individuals seeking healthcare, and is thus less likely to detect chronic brucellosis cases. It has been estimated that 40% of brucellosis cases may persist with chronic disease for two years following disease onset, and 10% may persist with clinical manifestations after six years^51^. Further investigations of the combined health impacts of acute and chronic human brucellosis in this population are warranted to evaluate the combined burden of disease^52^.

Risk factors associated with brucellosis in the univariable models in this study included younger age, male sex and herding livestock. Lasso regression selected herding livestock and age variables, which were retained for the multivariable model. The fact that the sex variable was not retained in the final model is likely to be due to correlation between male sex and having herded livestock. Higher risk of *Brucella* spp. exposure at an early age has been recognised for nomadic groups, with adults often more likely suffering from chronic brucellosis infection^2^. Within the largely Maasai community of the NCA, young boys are preferentially given responsibility for herding livestock (RFB personal communication during NCA community meetings). Herding can involve multiple activities that may put individuals at higher risk of exposure to *Brucella* spp. as well as many other zoonotic pathogens. These activities may include: frequent contact with livestock, assisting in animal parturition, butchering livestock and consuming specific organs raw or eating undercooked meat and blood^53^, and drinking raw milk directly from animals in the herd or flock^53^. Some of the more common risk factors for human brucellosis reported in East Africa such as: consumption of raw animal products^27,54^, assisting in animal parturition events and contact with aborted animal materials^31,55,56^ and slaughtering^57^ were not identified in this study. The small number of brucellosis cases identified and the high proportions of the study population participating in perceived high risk activities, complicate efforts to disentangle the relative importance of specific risk activities and transmission pathways in this population. Further investigation into activities conducted whilst herding that may increase exposure to *Brucella* spp. may identify additional specific risk factors for infection in the young herders identified as high risk in our study.

Diagnosis of brucellosis by clinical symptoms alone is unreliable due to varying clinical manifestations of infection^10^. In the present study brucellosis case status was positively associated with night sweats but negatively associated with back pain. These findings reinforce the challenges of using clinical symptoms for brucellosis case identification. In spite of this, brucellosis was a relatively common presumptive diagnosis made by clinicians at the Endulen Hospital, indicating high clinical awareness of the disease in this setting. There was also a significant association between presumptive diagnosis of brucellosis and brucellosis case status. Not all participants with a presumptive diagnosis of brucellosis were started on a brucellosis consistent treatment on the day of hospital presentation. However, the data presented on treatments refer to treatments prescribed at initial presentation only and do not capture later decisions based on additional findings, including the subsequent blood culture results provided through the study. Overall, these data highlight the challenges faced by clinicians in areas where brucellosis is endemic but access to high quality diagnostics including blood culture and SAT serology is limited. There is a need for improved diagnostic tools and diagnostic guidelines for the management of acute brucellosis and febrile illness more generally in Tanzania, and East Africa more widely^11^.

Brucellosis has been identified as one of six priority zoonotic diseases in Tanzania^58^, prompting the development of a national strategy for brucellosis prevention and control in humans and animals^59^. These study findings, including human prevalence estimates, identification of both *B. melitensis* and *B. abortus* as causes of human illness and risk factors for acute human illness, can inform the development of evidence-based control strategies for brucellosis in Tanzania.

## Conclusions

We found that brucellosis was the cause of 6.1% of febrile illness among study participants presenting for outpatient hospital care, and that *Brucella* spp. are the most commonly identified bloodstream infection in this predominantly pastoralist community from the NCA, Tanzania. Our findings show that human brucellosis is caused by both *B. melitensis* and *B. abortus* consistent with transmission from multiple livestock host species. Within this pastoralist community, young livestock herders are at high risk of brucellosis infection. Brucellosis control activities in Tanzania which focus on prevention of transmission to individuals at high risk of infection, and livestock vaccination campaigns directed at both cattle and small ruminants have potential for substantial impacts in reducing human brucellosis risk.

## Supplementary information

Supplementary Information.

## Data Availability

The datasets generated during and/or analysed during the current study are available in the Enlighten research data repository of the University of Glasgow (http://dx.doi.org/10.5525/gla.researchdata.978). The sequence typing data for the isolates from this study are available in the *Brucella MLST Databases* https://pubmlst.org/brucella/.
