# Supplementary material for: Prevalence and speciation of brucellosis in febrile patients from a pastoralist community of Tanzania
Source: Sci Rep. 2020 Apr 27;10:7081. doi: 10.1038/s41598-020-62849-4 (PMC7184621; doi:10.1038/s41598-020-62849-4)
Supplement: Supplementary file 1 — Supplementary Information. [file 41598_2020_62849_MOESM1_ESM.pdf]

Prevalence and speciation of brucellosis in febrile patients from a pastoralist community of Tanzania

Authors

Rebecca F. Bodenham[1], AbdulHamid S. Lukambagire[2], Roland T. Ashford[3], Joram J. Buza[4], Shama Cash-Goldwasser[5,6], John A. Crump[5-10], Rudovick R. Kazwala[2], Venance P. Maro[6,9], John McGiven[3], Nestory Mkenda[11], Blandina T. Mmbaga[5-7,9], Matthew P. Rubach[5,6,10,12], Philoteus Sakasaka[7], Gabriel M. Shirima[4], Emanuel S. Swai[13], Kate M. Thomas[7,8], Adrian M. Whatmore[3], Daniel T. Haydon[1], Jo E. B. Halliday[1]*

Email: *jo.halliday@glasgow.ac.uk

Affiliations

1: Institute of Biodiversity, Animal Health & Comparative Medicine, College of Medical Veterinary and Life Sciences, University of Glasgow, Glasgow, UK; 2: Sokoine University of Agriculture, Morogoro, Tanzania; 3: OIE/FAO Brucellosis Reference Laboratory, Department of Bacteriology, Animal & Plant Health Agency, Surrey, UK; 4: Nelson Mandela African Institution for Science and Technology, Arusha, Tanzania; 5: Duke Global Health Institute, Duke University, Durham, North Carolina, USA; 6: Kilimanjaro Christian Medical Centre, Moshi, Tanzania; 7: Kilimanjaro Clinical Research Institute, Moshi, Tanzania; 8: Centre for International Health, University of Otago, Dunedin, New Zealand ; 9: Kilimanjaro Christian Medical University College, Moshi, Tanzania; 10: Division of Infectious Diseases and International Health, Duke University Medical Center, North Carolina, USA; 11: Endulen Hospital, Ngorongoro Conservation Area, Tanzania; 12: Programme in Emerging Infectious Diseases, Duke-NUS Medical School, Singapore, Singapore; 13: Directorate of Veterinary Services, Ministry of Livestock and Fisheries, Dodoma, Tanzania

Supplementary Methods: Study Participant Questionnaire

5726256092

ZELS BRUCELLA - INDIVIDUAL QUESTIONNAIRE

SECTION 1: INTERVIEW DETAILS

1.1 Location ID

Z B L - ☐ - ☐☐☐☐

1.2 Enrollment Date (dd/mm/yyyy)

☐☐ / ☐☐ / ☐☐☐☐

1.3 Interviewer's initials

☐☐☐

HH Q ID

☐☐☐

1.4 Individual ID

Z B P - ☐☐☐☐

1.5 Interview Date (dd/mm/yyyy)

☐☐ / ☐☐ / ☐☐☐☐

Review Date (dd/mm/yyyy)

☐☐ / ☐☐ / ☐☐☐☐

Reviewer's initials

☐☐☐

1.6 Language

○ Kiswahili ○ English ○ Maasai

1.7 Primary informant

○ Self ○ Parent/Guardian ○ Relative ○ Other

SECTION 2: INDIVIDUAL DESCRIPTION

2.1 Sex **Jinsia**

○ Male ○ Female

2.2 Date of birth (dd/mm/yyyy) **Tarehe ya kuzaliwa**

☐☐ / ☐☐ / ☐☐☐☐

If only the year of birth is known, record 01 for dd and 07 for mm.
If year of birth is known, ask question 2.4.
Kama ni mwaka wa kuzaliwa pekee unajulikana jaza 01 (dd) na 07 (mm)
Kama mwaka wa kuzaliwa unajulikana, uliza swali 2.4

2.3 Age Class

○ 0-5 yr ○ 6-12 yr ○ 13-18 yr ○ 19-34 yr ○ 35-54 yr ○ > 55 yr

2.4 What is your tribe? **Kabila lako?**

○ Arusha ○ Maasai
○ Barabaig ○ Pare
○ Chagga ○ Sambaa
○ Iraqw ○ Other (specify)

☐☐☐☐☐☐☐☐☐☐☐☐☐

If Maasai or Arusha ask 2.5a/2.5b, otherwise proceed to 2.6
Kama ni Mmasai au Mwarusha uliza 2.5a/2.5b, vinginevyo nenda 2.6

2.5a. What is your ageset (men)?
Wewe ni rika gani (wanaume)?

○ Nyangulo
○ Ilkiponi/Korrianga
○ Ilkumunyak/ILandis (Ilkidotu)
○ Ilkishumu/ Makaa (Irkishomo)
○ ISeuri
○ Ilnyankusi/Meshuki

2.5b. What is your ageset (women)?
(these are 'unofficial' but commonly used):
Wewe ni rika gani (wanawake)?

○ Boda boda/Ingoipila: under 20yrs
○ Njujulai: 20-30yrs
○ Maharage (Intiamaragi)/N'gali: 30-36yrs
○ Mosogiro: 37-46yrs
○ Isusan: 47-56yrs
○ Ingaimuk (Ingaimug): 56-65yrs
○ Enderito: 65+yrs

2.6 What is your marital status? **Hali ya mahusiano ya ndoa?**

○ Married (Nimeolewa/oa)
○ Single (Sijaolewa/oa)
○ Divorced/separated (Nimeachika/acha)
○ Widowed (Miane)

2.7 Arusha District/ **Wilaya ya Arusha**

○ Arusha Rural ○ Ngorongoro
○ Arusha Urban ○ Meru
○ Longido ○ Karatu
○ Monduli ○ Other

Other Region/ Mkoa mwingine

☐☐☐☐☐☐☐☐☐☐☐☐☐☐

Other District/ Wilaya nyingine

☐☐☐☐☐☐☐☐☐☐☐☐☐☐

2.8 Ward **Kata**

☐☐☐☐☐☐☐☐☐☐☐☐☐☐

2.9 Village **Kijiji**

☐☐☐☐☐☐☐☐☐☐☐☐☐☐

2.10 Sub-village **Kitongoji** *(leave blank if none)*

☐☐☐☐☐☐☐☐☐☐☐☐☐☐

2.11. How long has your boma been in your current village?
Ni kwa muda gani boma lako lipo katika kijiji hiki?

○ Years **Miaka** ○ Months **Miezi** ○ Days **Siku**

☐☐☐

2.12 How many adults live in your boma?

☐☐ **Je, ni watu wazima wangapi wanaishi katika boma lako?**
(age 18 years or older/ miaka 18 au zaidi)

2.13 How many children live in your boma?

☐☐ **Je, ni watoto wangapi wanaishi katika boma lako?**
(age less than 18 years/ chini ya miaka 18)

2.14. In the past 30 days, have you travelled outside your home region?
Katika kipindi cha siku 30 zilizopita umesha safiri nje ya mkoa wako unaoishi?

If yes, provide details of the locations visited in the box below
Kama ndiyo, jaza katika kisanduku maeneo aliyo tembelea

○ Yes ○ No

☐

☐☐☐☐☐☐

2.15 How many years of education have you had?
Umepata elimu (darasani) kwa miaka mingapi?

- ○ No education (Sijasoma)
- ○ Primary (1-7 years) (Msingi)
- ○ Secondary (8-11 years) (Sekondari)
- ○ High school (12-13 years) (Sekondari ya juu)
- ○ University/college (Chuo kikuu/chuo)

2.16 What are your main work/professional activities?
Ajira yako kuu ni ipi?

	Primary occupation **Kazi ya kudumu** (Choose one) (Chagua moja)	Other occupation **Kazi ya ziada** (Choose many) (Chagua yote yanayohusika)
livestock attendant 1 **mchungi/mfugaji**	○	○
butcher/abattoir worker 2 **mchinjaji/mfanyakazi wa machinjioni**	○	○
wildlife worker 3 **mfanyakazi wa wanyamapori**	○	○
milk supplier 4 **msambazaji wa maziwa**	○	○
student 5 **mwanafunzi**	○	○
crafts person 6 **mhunzi**	○	○
veterinarian 7 **mganga wa mifugo**	○	○
housewife 8 **mama wa nyumbani**	○	○
office worker 9 **mfanyakazi wa ofisini**	○	○
healthcare worker 10 **mhudumu wa afya**	○	○
merchant/trader 11 **mjasiriamali/ mfanyabiashara**	○	○
teacher 12 **mwalimu**	○	○
driver 13 **dereva**	○	○
sewer worker 14 **wazibua vyoo**	○	○
guard/police 15 **askari/ polisi**	○	○
unemployed 16 **sijaajiriwa**	○	○
pre-working age 17 **bado hajafikia umri wa kufanya kazi**	○	○
other 18 **nyingineo**	○	

SECTION 3: CURRENT AND RECENT ILLNESS

3.1 During the past two weeks, have you had any of the following types of illness? (indicate all that apply)
Katika wiki mbili zilizopita, umewahi kupata yoyote kati ya magonjwa yafuatayo? (ainisha yote yanayohusika)

Diarrhoea/ **kuharisha**	○ Yes	○ No
Respiratory illness/ **magonjwa ya kifua/ kupumua**	○ Yes	○ No
Fever/ **homa**	○ Yes	○ No

diarrhoea: >= 3 loose stools within a 24 hours period
kuharisha: >= choo laini 3 ndani ya muda wa saa 24
respiratory illness: cough or difficulty breathing
magonjwa ya kifua/kupumua: kuhohoa au matatizo ya kupumua
fever: report of fever

If yes to fever in 3.1,ask questions 3.2 - 3.5
Kama jibu la homa ni ndio kwa swali 3.1, tafadhali uliza maswali 3.2 - 3.5

3.2 Is your fever continual or intermittent?
Je, homa yako ni ya mfululizo au ya vipindi?

○ Continual **mfululizo** ○ Intermittent **vipindi**

3.3 How long ago did the fever start?
Je, ni muda gani tangu kuanza kwa homa?

○ Days **siku** ○ Months **miezi** ○ Years **miaka**

3.4 How long ago did your illness start?
Je, ni muda gani ugonjwa wako ulikuanza?

○ Days **siku** ○ Months **miezi** ○ Years **miaka**

3.5 Have you had any of the following signs or symptoms of illness during this current illness?
Umepata yoyote kati ya dalili zifuatazo za ugonjwa katika kipindi hiki cha kuugua kwako?

If not currently febrile, please ask for past 2 weeks and past 12 months only
Kama hana homa kwa sasa, uliza katika kipindi cha majuma mawili yaliyopita na miezi 12 iliyopita tu

	**Current Illness**	**Past 2 weeks**	**Past 12 months**
night sweats **jasho la usiku**	○ Yes ○ No ○ DK	○ Yes ○ No ○ DK	○ Yes ○ No ○ DK
fatigue **uchovu wa mwili**	○ Yes ○ No ○ DK	○ Yes ○ No ○ DK	○ Yes ○ No ○ DK
rigors **kutetemeka baridi**	○ Yes ○ No ○ DK	○ Yes ○ No ○ DK	○ Yes ○ No ○ DK
jaundice/yellow eyes **manjano/macho ya njano**	○ Yes ○ No ○ DK	○ Yes ○ No ○ DK	○ Yes ○ No ○ DK
joint pain **maumivu ya viungo**	○ Yes ○ No ○ DK	○ Yes ○ No ○ DK	○ Yes ○ No ○ DK
swollen joints **kuvimba kwa viungo**	○ Yes ○ No ○ DK	○ Yes ○ No ○ DK	○ Yes ○ No ○ DK
muscle pain **maumivu ya misuli**	○ Yes ○ No ○ DK	○ Yes ○ No ○ DK	○ Yes ○ No ○ DK
back pain **maumivu ya mgongo**	○ Yes ○ No ○ DK	○ Yes ○ No ○ DK	○ Yes ○ No ○ DK
headache **kuumwa kichwa**	○ Yes ○ No ○ DK	○ Yes ○ No ○ DK	○ Yes ○ No ○ DK
stiff neck **shingo kukakamaa**	○ Yes ○ No ○ DK	○ Yes ○ No ○ DK	○ Yes ○ No ○ DK
convulsions **degedege**	○ Yes ○ No ○ DK	○ Yes ○ No ○ DK	○ Yes ○ No ○ DK
conjunctival suffussion **macho kua mekundu**	○ Yes ○ No ○ DK	○ Yes ○ No ○ DK	○ Yes ○ No ○ DK
ear pain or drainage **maumivu ya sikio**	○ Yes ○ No ○ DK	○ Yes ○ No ○ DK	○ Yes ○ No ○ DK
sore throat **kuumwa koo**	○ Yes ○ No ○ DK	○ Yes ○ No ○ DK	○ Yes ○ No ○ DK
loss of appetite **kupoteza hamu ya kula**	○ Yes ○ No ○ DK	○ Yes ○ No ○ DK	○ Yes ○ No ○ DK
abdominal pain **maumivu ya tumbo**	○ Yes ○ No ○ DK	○ Yes ○ No ○ DK	○ Yes ○ No ○ DK
constipation **kuvimbiwa**	○ Yes ○ No ○ DK	○ Yes ○ No ○ DK	○ Yes ○ No ○ DK
bloody stool **choo chenye damu**	○ Yes ○ No ○ DK	○ Yes ○ No ○ DK	○ Yes ○ No ○ DK
vomiting **tapika**	○ Yes ○ No ○ DK	○ Yes ○ No ○ DK	○ Yes ○ No ○ DK
dysuria **unapata maumivu unapokojoa**	○ Yes ○ No ○ DK	○ Yes ○ No ○ DK	○ Yes ○ No ○ DK
swollen painful testicles (male only) **Kuvimba korodani/ makende (wanaume)**	○ Yes ○ No ○ DK	○ Yes ○ No ○ DK	○ Yes ○ No ○ DK
rash **upele**	○ Yes ○ No ○ DK	○ Yes ○ No ○ DK	○ Yes ○ No ○ DK
eschar **kovu**	○ Yes ○ No ○ DK	○ Yes ○ No ○ DK	○ Yes ○ No ○ DK

For the next question:
Hospital participants should complete question.3.6a (and 3.6b if appropriate) but not 3.7a /3.7b.
Households participants should complete question 3.7a (and 3.7b if appropriate) but not 3.6a/ 3.6b
All participants should complete questions 3.8 onwards

3.6a Have you sought care for this illness at another location prior to presentation at Endulen?
Je, ulishatafuta tiba ya ugonjwa huu sehemu nyingine kabla ya Endulen? ○ Yes ○ No *If yes, how many locations Kama ndio, sehemu ngapi?* ☐☐

If yes for question 3.6a ask 3.6b/ Kama ndio kwa sawali 3.6a, uliza 3.6b

3.6b Which type of Healthcare facilities were visited?
Ni aina gani ya vituo vya afya ulivyohudhuria?

- ○ Pharmacy **(Duka la dawa)**
- ○ Dispensary **(Zahanati)**
- ○ Traditional healer **(Mganga wa jadi)**
- ○ Health center **(Kituo cha afya)**
- ○ Other Hospital ☐☐☐☐☐☐☐☐☐
- ○ Other ☐☐☐☐☐☐☐☐☐☐☐☐☐

<u>For febrile participants in the household only. Kwa wagonjwa wa homa katika kaya tu.</u>

3.7a Have you sought care for your illness with a healthcare provider?
Je, umesha tafuta tiba ya ugonjwa wako kwa mtoa huduma ya afya? ○ Yes ○ No *If yes, how many Kama ndio, wangapi?* ☐☐

If yes for question 3.7a ask 3.7b/ Kama ndio kwa sawali 3.7a, uliza 3.7b

3.7b Which type of Healthcare facilities were visited?
Ni aina gani ya vituo vya afya ulivyohudhuria?

- ○ Pharmacy **(Duka la dawa)**
- ○ Dispensary **(Zahanati)**
- ○ Traditional healer **(Mganga wa jadi)**
- ○ Health center **(Kituo cha afya)**
- ○ Other Hospital ☐☐☐☐☐☐☐☐☐
- ○ Other ☐☐☐☐☐☐☐☐☐☐☐☐☐

3.8 Have you taken any medicines over the past 2 weeks?
Umetumia dawa yoyote katika kipindi cha majuma 2 yaliyopita?

○ Yes ○ No ○ DK *If yes, what was the medication(s) for? Kama ndio, dawa zilikua kwa ajili gani?*

A. ☐☐☐☐☐☐☐☐☐☐☐☐☐
B. ☐☐☐☐☐☐☐☐☐☐☐☐☐
C. ☐☐☐☐☐☐☐☐☐☐☐☐☐
D. ☐☐☐☐☐☐☐☐☐☐☐☐☐

If yes, what was the medication(s) name(s)? Kama ndio, dawa hizo zinaitwaje?

A. ☐☐☐☐☐☐☐☐☐☐☐☐☐
B. ☐☐☐☐☐☐☐☐☐☐☐☐☐
C. ☐☐☐☐☐☐☐☐☐☐☐☐☐
D. ☐☐☐☐☐☐☐☐☐☐☐☐☐

☐☐☐☐☐☐

3.9 Have you taken any other medications over the past 2 weeks?
Umetumia dawa nyingine zozote katika kipindi cha majuma 2 yaliyopita?

Tuberculosis drugs/ **Dawa za kifua kikuu**	O Yes O No O DK
Analgesics/ **Dawa za kupunguza maumivu**	O Yes O No O DK
Antiretrovirals/ **Dawa za ARV**	O Yes O No O DK
Other/ **Nyinginezo**	O Yes O No O DK

SECTION 4: PREGNANCY HISTORY (females only)

We would now like to ask you some questions about your pregnancy history/
Tungependa sasa kukuuliza baadhi ya maswali kuhusu historia yako ya ujauzito

4.1 Are you comfortable answering questions about this?
Je, unajisikia vizuri kujibu maswali kuhusiana na hili?
O Yes O No

If yes, proceed to next questions. If no, skip to SECTION 5
Kama ndio, endelea na maswali yafuatayo. Kama hapana, nenda SEHEMU ya 5

4.2 Have you started menstruating?
Umeanza kupata siku za mwezi/ hedhi (umefikia kuvunja ungo)? O Yes O No

If no, skip to SECTION 5/
Kama ni hapana nenda SEHEMU ya 5.

4.3 Have you ever been pregnant?
Umewahi kupata ujauzito? O Yes O No
If no, skip to SECTION 5./
Kama hapana, nenda SEHEMU ya 5

4.4 How many times have you been pregnant (including abortions, miscarriages, stillbirths and live births)?

Ni mara ngapi umepata ujauzito (ikihusisha mimba zilizotolewa zilizoharibika, kuzaa watoto wafu na watoto hai)?

4.5 How many live births have you had?
Umejifungua mara ngapi watoto wakiwa hai?

4.6 Have you ever had a miscarriage or stillbirth?
Je, umewahi kupata mimba ikaharibika au kutoka yenyewe au kichanga kuzaliwa mfu? O Yes O No

If yes, how many?
Kama ndio, mara ngapi?

4.7 What was the outcome of your last pregnancy?
Je, yapi yalikua matokeo ya ujauzito wako wa mwisho?

O Miscarriage or spontaneous abortion (<28 weeks)
Mtoto kufia tumboni au mimba kuharibika (kabla ya wiki 28 za ujauzito)
O Fetal death or stillbirth (after 28 weeks)
Mtoto kufia tumboni/kuzaliwa mfu (baada ya wiki 28 za ujauzito)
O Live birth
Mtoto aliye hai
O NA- first pregnancy ongoing
Bado ni mjamzito
O Other **Nyingine**

SECTION 5: BRUCELLOSIS HISTORY

5.1 Before we talked to you about this study, had you heard of a disease called brucellosis/ brucella?
Kabla ya kuzungumza na wewe kuhusu huu utafiti, je uliwahi kusikia ugonjwa unaoitwa brucellosis/ brusela? O Yes O No

If no to 5.1, skip to 5.9 / Kama hapana kwa swali 5.1, nenda swali la 5.9

5.2 Can you tell us what are the usual symptoms of brucellosis in people?
Unaweza kutuambia dalili za mtu mwenye brucellosis?

Go through the list of symptoms/ signs and prompt the respondent to find out if they think each is associated with brucellosis. Record a Yes (Y) or No (N) response after prompting. record any additional reported signs or symptoms in the text box.
Pitia orodha ya dalili/ viashiria na muulize mshiriki kama dalili/ viashiria hivi vina uhusiano na brucellosis. Jaza jibu la Yes (Y) au No (N) baada ya kuuliza na jaza dalili zozote nyingine kwenye kisanduku mwishoni mwa jedwali.

I don't know/ **siiui**	O DK
malaise/ **uchovu**	O Yes O No
headache/ **maumivu ya kichwa**	O Yes O No
fever/ **homa**	O Yes O No
abdominal pain/ **maumivu ya tumbo**	O Yes O No
anorexia/ **kushindwa kula**	O Yes O No
joint pain/ **maumivu ya viungo**	O Yes O No
back pain/ **maumivu ya mgongo**	O Yes O No
sweats/ **kutokwa na jasho**	O Yes O No
chills/ **kutetemeka baridi**	O Yes O No
chest pain/ **maumivu ya kifua**	O Yes O No
muscle aches/ **kuumwa kwa misuli**	O Yes O No
cough/ **kukohoa**	O Yes O No
constipation/ **kufunga choo au kuvimbiwa**	O Yes O No
neck pain/ **maumivu ya shingo**	O Yes O No
diarrhoea/ **kuharisha**	O Yes O No
vomiting/ **kutapika**	O Yes O No
breathlessness/ **kupumua kwa shida**	O Yes O No
weight loss/ **kupungua kwa uzito**	O Yes O No
joint swelling/ **kuvimba kwa viungo**	O Yes O No
rash/ **upele**	O Yes O No
orchitis (in males)/ **kuvimba korodani**	O Yes O No
Other signs/ symptoms/ **Dalili/ viashiria vingine:**	

5.3 Have you ever been diagnosed with brucellosis?
Je, ulishawahi kugundulika una brucellosis?

○ Yes ○ No

If no to 5.3, skip to 5.8/ Kama hapana kwa 5.3, nenda 5.8

5.4 When was the diagnosis made? / **Je, uligundulika lini?**

Month/ **Mwezi** Year/ **Mwaka**

☐☐ / ☐☐☐☐

5.5 Where was the diagnosis made? / **Je, uligundulika wapi?**

5.6 Did you receive treatment? /**Je, ulipatiwa matibabu?**

○ Yes ○ No

If no to 5.6, skip to 5.8/ Kama hapana kwa swali la 5.6, nenda 5.8

5.7 What was your treatment? / **Ulipatiwa tiba ipi?**

5.8 Have any other members of your family ever been diagnosed with brucellosis?
Je, kuna mtu yeyote katika kaya yako aliwahi kugundulika ana brucellosis?

○ Yes ○ No

If yes, record who in the family, where and by who the diagnosis was made?
Kama ndio, jaza ni nani aliyegundulika katika familia na ni wapi na ni nani aliye gundua?

If participant is febrile.
Kama mshiriki ana homa.

5.9 Do you know anyone in your boma or village who has current symptoms similar to yours?
Je, unamjua mtu yeyote katika boma au kijiji chako mwenye dalili sawa na za kwako?

○ Yes ○ No

If yes, fill out ***"contact screening form"****./*
Kama ndio, jaza fomu ya ***"contact screening"***

		Number of people **Idadi ya watu**
Boma member **Mwanaboma**	○ Yes ○ No ○ DK	☐☐
Village member **Mwanakijiji**	○ Yes ○ No ○ DK	☐☐

SECTION 6: FOOD

6.1 In the past 30 days, have you consumed the following types of boiled or pasteurised dairy products?
Katika siku 30 zilizopita, umetumia/ kula aina za mazao ya maziwa zilizochemshwa au za viwandani?

If yes, include how many days per week (in a typical week).
If no, ask if the dairy product has been consumed in the past 12 months

Kama ndiyo, jaza ni siku ngapi katika juma (juma la kawaida)
Kama hapana, uliza kama aina hiyo ya mazao ya maziwa alitumia katika kipindi cha miezi 12 iliyopita

	**Past 30 days**	**Number of days (1-7)**	**Past 12 months**
Milk **Maziwa**	○ Yes ○ No	☐	○ Yes ○ No
Yogurt **Maziwa mtindi**	○ Yes ○ No	☐	○ Yes ○ No
Cheese **Jibini**	○ Yes ○ No	☐	○ Yes ○ No
Butter **Siagi**	○ Yes ○ No	☐	○ Yes ○ No
Cream **Mafuta ya maziwa**	○ Yes ○ No	☐	○ Yes ○ No
Other food (e.g. uji, ndizi) prepared by adding dairy products before or during cooking **Vyakula vingine (mfano uji, ndizi) kwa kuviongezea mazao ya maziwa kabla au wakati wakupikwa**	○ Yes ○ No	☐	○ Yes ○ No
Other food (e.g. uji, ndizi) prepared by adding pasteurised or boiled dairy products after cooking **Vyakula vingine (mfano uji, ndizi) kwa kuviongezea mazao ya maziwa yaliyochemshwa baada ya kupika**	○ Yes ○ No	☐	○ Yes ○ No
Other products made from boiled or pasteurised dairy products **Mazao mengine yatokanayo na maziwa ya viwandani au maziwa yaliyochemshwa** ☐☐☐☐☐☐☐☐☐☐☐☐☐	○ Yes ○ No	☐	○ Yes ○ No
cheese, butter, cream or yogurt but unsure whether milk raw or boiled/ pasteurised **Jibini, siagi, mafuta au mgando/mtindi lakini hakuna uhakika kama maziwa yalikuwa mabichi, yaliyochemshwa au ya kiwandani**	○ Yes ○ No	☐	○ Yes ○ No

6.2 Which animals did the boiled or pasteurised milk products come from? (Choose all that apply and prompt all options)
Je, ni kutoka kwa wanyama wepi maziwa yaliyochemshwa au bidhaa za maziwa ya kiwandani yamepatikana?
(Chagua yote yanayohusika)

cow/ **ng'ombe wako**	○ Yes ○ No
goat/ **mbuzi wako**	○ Yes ○ No
sheep/ **kondoo wako**	○ Yes ○ No
other animal/ **wengine**	○ Yes ○ No
☐☐☐☐☐☐☐☐☐☐	

☐☐☐☐☐☐

6.3 In the past 30 days, have you consumed any of the following types of raw meat or offal or raw animal blood?
Katika kipindi cha siku 30 zilizopita, je wewe umetumia/kula yoyote kati ya aina ya nyama mbichi au nyama za ndani, au damu mbichi ya mnyama?

If yes, include how many days per week (in a typical week).
If no, ask if the meat/offal/blood has been consumed in the past 12 months.

Kama ndio, uliza ni siku ngapi katika juma (juma la kawaida)
Kama hapana, uliza kama nyama mbichi/nyama za ndani/ damu mbichi ilitumika miezi 12 iliyopita

	Past 30 days	Number of days (1-7)	Past 12 months
raw cow blood<br>**damu mbichi ya ng'ombe**	O Yes O No	☐	O Yes O No
raw goat blood<br>**damu mbichi ya mbuzi**	O Yes O No	☐	O Yes O No
raw sheep blood<br>**damu mbichi ya kondoo**	O Yes O No	☐	O Yes O No
raw blood from other animal<br>**damu mbichi ya mnyama mwingine**	O Yes O No	☐	O Yes O No
☐☐☐☐☐☐☐☐☐☐☐☐☐			
raw meat or offal from cow<br>**nyama mbichi au nyama za ndani kutoka kwa ng'ombe**	O Yes O No	☐	O Yes O No
raw meat or offal from goat<br>**nyama mbichi au za ndani kutoka kwa mbuzi**	O Yes O No	☐	O Yes O No
raw meat or offal from sheep<br>**nyama mbichi au za ndani kutoka kwa kondoo**	O Yes O No	☐	O Yes O No
raw meat or offal from another animal<br>**nyama mbichi au za ndani kutoka kwa mnyama mwingine**	O Yes O No	☐	O Yes O No
☐☐☐☐☐☐☐☐☐☐☐☐☐			

6.4 In the past 30 days have you consumed soup with blood?
Je, katika kipindi cha siku 30 zilizopita uliwahi kunywa supu yenye damu (kisusio)? O Yes O No

If yes, blood from which animal?
Kama ndio, ni damu kutoka kwa mnyama yupi?

Cow/ **Ng'ombe**	O Yes O No
Goat/ **Mbuzi**	O Yes O No
Sheep/ **Kondoo**	O Yes O No
Other/ **Wengine**	O Yes O No
☐☐☐☐☐☐☐☐☐☐	

6.5 In the past 30 days have you consumed blood mixed with milk?
Je, katika kipindi cha siku 30 zilizopita uliwahi kunywa maziwa yaliyochanganywa na damu (mlaso)? O Yes O No

If yes, blood from which animal?
Kama ndio, ni damu kutoka kwa mnyama yupi?

Cow/ **Ng'ombe**	O Yes O No
Goat/ **Mbuzi**	O Yes O No
Sheep/ **Kondoo**	O Yes O No
Other/ **Wengine**	O Yes O No
☐☐☐☐☐☐☐☐☐☐	

If yes, milk from which animal?
Kama ndio, ni maziwa kutoka kwa mnyama yupi?

Cow/ **Ng'ombe**	O Yes O No
Goat/ **Mbuzi**	O Yes O No
Sheep/ **Kondoo**	O Yes O No
Other/ **Wengine**	O Yes O No
☐☐☐☐☐☐☐☐☐☐	

6.6 In the past 12 months, have you consumed meat or offal from an aborted animal or the placenta of an aborted animal?
Katika miezi 12 iliyopita, umetumia/kula nyama au nyama za ndani kutoka kwa kichanga cha mnyama au kondo la nyuma? O Yes O No

If no, skip to 6.7
If yes, complete table below. If activity was performed in past 30 days, include how many days per week (in a typical week).

Kama hapana, nenda swali 6.7
Kama ndio, kamilisha jedwali lifuatalo. Kama shughuli ilifanyika katika siku 30 zilizopita jaza ni siku ngapi katika juma (juma la kawaida)

	Past 30 days	Number of days (1-7)	Past 12 months
Cow / N**g'ombe**	O Yes O No	☐	O Yes O No
Goat/ **Mbuzi**	O Yes O No	☐	O Yes O No
Sheep/ **Kondoo**	O Yes O No	☐	O Yes O No
Another animal<br>**Mnyama mwingine**	O Yes O No	☐	O Yes O No
☐☐☐☐☐☐☐☐☐☐☐☐			

6.7 Was the meat or offal raw?
Je, nyama/ nyama za ndani zilikua mbichi? O Yes O No

6.8 In the past 30 days, have you consumed the following types of raw dairy products?
Katika siku 30 zilizopita, umetumia/ kula aina za mazao ya maziwa mabichi zifuatazo?

If yes, include how many days per week (in a typical week).
If no, ask if the raw dairy product has been consumed in the past 12 months

Kama ndiyo, jaza ni siku ngapi katika juma (juma la kawaida)
Kama hapana, uliza kama aina hiyo ya mazao ya maziwa mabichi yalitumika katika kipindi cha miezi 12 iliyopita

	Past 30 days	Number of days (1-7)	Past 12 months
Milk **Maziwa**	O Yes O No	☐	O Yes O No
Yogurt **Maziwa mtindi**	O Yes O No	☐	O Yes O No
Cheese **Jibini**	O Yes O No	☐	O Yes O No
Butter **Siagi**	O Yes O No	☐	O Yes O No
Cream **Mafuta ya maziwa**	O Yes O No	☐	O Yes O No
Other food (e.g. uji, ndizi) prepared by adding raw dairy products before or during cooking **Vyakula vingine (mfano uji, ndizi) kwa kuongeza mazao ya maziwa mabichi kabla au wakati unapika**	O Yes O No	☐	O Yes O No
Other food (e.g. uji, ndizi) prepared by adding raw dairy products after cooking **Vyakula vingine (mfano uji, ndizi) kwa kuongeza mazao ya maziwa mabichi baada ya kupika**	O Yes O No	☐	O Yes O No
Other products made from other raw dairy products **Mazao mengine yatokanayo katika mazao mengine ya maziwa mabichi**	O Yes O No	☐	O Yes O No

6.9 If you do not tend to drink raw milk, or consume it in other dairy or food products, are there any particular circumstances where you might consume raw milk or it's products?
Kama huna nia ya kunywa maziwa mabichi au kuyatumia katika mazao yoyote ya maziwa au vyakula, je kuna mazingira yoyote huenda yakasababisha kunywa maziwa mabichi au mazao yake?

O Yes O No *If yes, under what circumstances?* *Kama ndio, ni katika mazingira gani?*

SECTION 7:ANIMAL RELATED ACTIVITIES

7.1 Have you milked any animals in the past 12 months?
Je wewe umekamua wanyama wowote katika kipindi cha miezi 12 iliyopita?
O Yes O No

If no, skip to 7.2
If yes, complete the following table. If activity was performed in past 30 days, include how many days per week (in a typical week).

Kama hapana nenda swali 7.2
Kama ndio, kamilisha jedwali lifuatalo. Kama shughuli ilifanyika katika siku 30 zilizopita jaza ni siku ngapi katika juma (juma la kawaida)

	Past 30 days	Number of days (1-7)	Past 12 months
cattle **ng'ombe**	O Yes O No	☐	O Yes O No
goats **mbuzi**	O Yes O No	☐	O Yes O No
sheep **kondoo**	O Yes O No	☐	O Yes O No
another animal **wengineo**	O Yes O No	☐	O Yes O No

7.2 In the past 12 months, have you slept in the same room or enclosure as any animals?
Katika miezi 12 iliyopita, umelala usiku kucha katika chumba kimoja au sehemu moja na wanyama wowote?
O Yes O No

If no, skip to 7.3
If yes, complete table below. If activity was performed in past 30 days, include how many days per week (in a typical week).
Kama hapana nenda swali 7.3
Kama ndio, kamilisha jedwali lifuatalo. Kama shughuli ilifanyika katika siku 30 zilizopita jaza ni siku ngapi katika juma (juma la kawaida)

	Past 30 days	Number of days (1-7)	Past 12 months
cattle **ng'ombe**	O Yes O No	☐	O Yes O No
goats **mbuzi**	O Yes O No	☐	O Yes O No
sheep **kondoo**	O Yes O No	☐	O Yes O No
another animal **wengineo**	O Yes O No	☐	O Yes O No

7.3 In the past 12 months have you handled the waste (manure) of any animals, including during building construction, cleaning animal pens, use as fertiliser etc.?
Katika kipindi cha miezi 12 iliyopita umeshashika samadi ya mnyama yeyote ikiwemo katika ujenzi, usafi wa zizi, matumizi ya mbolea n.k?
O Yes O No

If no, skip to7.4
If yes, complete table below. If activity was performed in past 30 days, include how many days per week (in a typical week).
Kama hapana nenda swali 7.4
Kama ndio, kamilisha jedwali lifuatalo. Kama shughuli ilifanyika katika siku 30 zilizopita jaza ni siku ngapi katika juma (juma la kawaida)

	Past 30 days	Number of days (1-7)	Past 12 months
cattle **ng'ombe**	O Yes O No	☐	O Yes O No
goats **mbuzi**	O Yes O No	☐	O Yes O No
sheep **kondoo**	O Yes O No	☐	O Yes O No
another animal **wengineo**	O Yes O No	☐	O Yes O No

7.4 Have you herded or used any animals for herding in the past 12 months?
Je, wewe umechunga au kumtumia yoyote kati ya wanyama kwa ajili ya kuchungia katika miezi 12 iliyopita?
O Yes O No

If no, skip to 7.5
If yes, complete table below. If activity was performed in past 30 days, include how many days per week (in a typical week).
Kama hapana nenda swali 7.5
Kama ndio, kamilisha jedwali lifuatalo. Kama shughuli ilifanyika katika siku 30 zilizopita jaza ni siku ngapi katika juma (juma la kawaida)

	**Past 30 days**	**Number of days (1-7)**	**Past 12 months**
cattle **ng'ombe**	○ Yes ○ No		○ Yes ○ No
goats **mbuzi**	○ Yes ○ No		○ Yes ○ No
sheep **kondoo**	○ Yes ○ No		○ Yes ○ No
dogs **mbwa**	○ Yes ○ No		○ Yes ○ No
another animal **mnyama mwingine**	○ Yes ○ No		○ Yes ○ No

7.5 Have you assisted with the birthing of any animals in the past 12 months?
Je, ulisaidia kuzalisha mnyama yeyote katika miezi 12 iliyopita?

○ Yes ○ No

If yes, number of animals in past 30 days
If no, ask if they have assisted with birthing of any of these animals in the past 12 months
Kama ndio, idadi ya wanyama katika kipindi cha siku 30 zilizopita
Kama hapana, uliza kama walisaidia kuzalisha wanyama wowote katika miezi 12 iliyopita

	**Past 30 days**	**Number of animals**	**Past 12 months**
cattle **ng'ombe**	○ Yes ○ No		○ Yes ○ No
goats **mbuzi**	○ Yes ○ No		○ Yes ○ No
sheep **kondoo**	○ Yes ○ No		○ Yes ○ No
donkeys **punda**	○ Yes ○ No		○ Yes ○ No
another animal **mnyama mwingine**	○ Yes ○ No		○ Yes ○ No

7.6 Have you handled/had contact with any placental or birth material of any animals in the past 12 months?
Je, umewahi kushika/kugusa kondo la nyuma au vitu vya uzazi vya mnyama yeyote katika miezi 12 iliyopita?

○ Yes ○ No

If no, skip to 7.7 If yes, complete table below.
Kama hapana, nenda swali 7.7
Kama ndio,kamilisha jedwali lifuatalo

	**Past 30 days**	**Number of animals**	**Past 12 months**
cattle **ng'ombe**	○ Yes ○ No		○ Yes ○ No
goats **mbuzi**	○ Yes ○ No		○ Yes ○ No
sheep **kondoo**	○ Yes ○ No		○ Yes ○ No
dogs **mbwa**	○ Yes ○ No		○ Yes ○ No
another animal **mnyama mwingine**	○ Yes ○ No		○ Yes ○ No

7.7 Have you handled/had contact with any aborted birth products from any animals in the past 12 months, including dead young/ offspring, animal fluid, placenta or blood?
Je, umewahi kushika/ kugusa vitu vyovyote vya mimba iliyotoka/ haribika ikiwemo kichanga mfu, maji maji ya mnyama, kondo la nyuma au damu kutoka kwa mnyama yoyote katika miezi 12 iliyopita?

○ Yes ○ No

If no, skip to 7.8 .If yes, complete table below.
Kama hapana, nenda swali 7.8 Kama ndio, kamilisha jedwali lifuatalo

	**Past 30 days**	**Past 12 months**
cattle/ **ng'ombe**	○ Yes ○ No	○ Yes ○ No
goats/ **mbuzi**	○ Yes ○ No	○ Yes ○ No
sheep/ **kondoo**	○ Yes ○ No	○ Yes ○ No
another animal/ **wengineo**	○ Yes ○ No	○ Yes ○ No

7.8 Have you slaughthered or butchered (or assisted in butchering) any livestock or domestic animals in the past 12 months?
Je wewe ulichinja au kukatakata (au kusaidia kuchinja au kukatakata) yeyote kati ya mifugo au wanyama wanaofugwa katika miezi 12 iliyopita?

○ Yes ○ No

If no, skip to 7.9. If yes, complete table below.
Kama hapana, nenda swali 7.9 Kama ndio, kamilisha jedwali lifuatalo

	**Past 30 days**	**Past 12 months**
cattle/ **ng'ombe**	○ Yes ○ No	○ Yes ○ No
goats/ **mbuzi**	○ Yes ○ No	○ Yes ○ No
sheep/ **kondoo**	○ Yes ○ No	○ Yes ○ No
pigs/ **nguruwe**	○ Yes ○ No	○ Yes ○ No
another animal/ **mwingine**	○ Yes ○ No	○ Yes ○ No

7.9 Have you handled/had contact with the carcass/ carcasses of any livestock or domestic animals in the past 12 months?
Je, umewahi kushika/ kugusa mzoga/ mizoga ya mifugo au wanyama wanaofugwa katika kipindi cha miezi 12 iliyopita?

○ Yes ○ No

If no, skip to 7.10 .If yes, complete table below.
Kama hapana, nenda swali 7.10. Kama ndio, kamilisha jedwali lifuatalo

	**Past 30 days**	**Number of animals**	**Past 12 months**
cattle **ng'ombe**	○ Yes ○ No		○ Yes ○ No
goats **mbuzi**	○ Yes ○ No		○ Yes ○ No
sheep **kondoo**	○ Yes ○ No		○ Yes ○ No
pigs **nguruwe**	○ Yes ○ No		○ Yes ○ No
dogs **mbwa**	○ Yes ○ No		○ Yes ○ No
donkeys **punda**	○ Yes ○ No		○ Yes ○ No
another animal **mnyama mwingine**	○ Yes ○ No		○ Yes ○ No

7.10 Have you handled/had contact with the carcass/ carcasses of any wild animals in the past 12 months?
Je, umewahi kushika/ kugusa mzoga/ mizoga ya wanyama wa wowote wa mwituni katika kipindi cha miezi 12 iliyopita? ○ Yes ○ No

If no, skip to 7.11 .If yes, complete table below.
Kama hapana, nenda swali 7.11.Kama ndio, kamilisha jedwali lifuatalo

	Past 30 days	Number of animals	Past 12 months
zebra **pundamilia**	○ Yes ○ No	☐	○ Yes ○ No
wildebeest **nyumbu**	○ Yes ○ No	☐	○ Yes ○ No
buffalo **nyati**	○ Yes ○ No	☐	○ Yes ○ No
antelope **swala**	○ Yes ○ No	☐	○ Yes ○ No
another animal **wengineo**	○ Yes ○ No	☐	○ Yes ○ No

7.11 Have you handled/prepared raw animal blood, meat or offal in the past 12 months?
Katika kipindi cha miezi 12 iliyopita umeshika/ andaa damu mbichi, nyama au nyama za ndani za wanyama? ○ Yes ○ No

If yes, complete the following table.If no, skip to next question.
Kama ndio, kamilisha jedwali lifuatalo. Kama hapana nenda swali linalofuata

	Past 30 days	Number of days (1-7)	Past 12 months
Cow **Ng'ombe**	○ Yes ○ No	☐	○ Yes ○ No
Goat **Mbuzi**	○ Yes ○ No	☐	○ Yes ○ No
Sheep **Kondoo**	○ Yes ○ No	☐	○ Yes ○ No
Another animal **Mnyama mwingine**	○ Yes ○ No	☐	○ Yes ○ No

7.12 Have you handled or prepared animal hides from any animal in the past 12 months?
Katika kipindi cha miezi 12 iliyopita umeshika au kuandaa ngozi za mnyama au wanyama wowote? ○ Yes ○ No

If yes, complete table below. If no, skip to next question.
Kama ndio, kamilisha jedwali lifuatalo. Kama hapana, uliza swali linalofuata.

	Past 30 days	Number of days (1-7)	Past 12 months
cow **ng'ombe**	○ Yes ○ No	☐	○ Yes ○ No
goat **mbuzi**	○ Yes ○ No	☐	○ Yes ○ No
sheep **kondoo**	○ Yes ○ No	☐	○ Yes ○ No
another animal **mwingineo**	○ Yes ○ No	☐	○ Yes ○ No

7.13 Have any of your family's livestock kept at your boma, aborted or delivered still-born offspring in the last 12 months?
Kuna mfugo yoyote (ng'ombe, kondoo au mbuzi) anayefugwa katika boma hili,ametoa/ameharibu mimba au amezaa mtoto mfu katika miezi 12 iliopita?

If yes, indicate the number of abortions/ stillbirths in the last 12 months.
Record NA if that species is not kept at this boma
Kama ndio, ainisha idadi ya mimba zilizoharibika/ watoto waliozaliwa wafu katika miezi 12 iliopita.
Jaza NA kama mnyama hafugwi katika boma hili

Cattle **(Ng'ombe)** ○ Yes ○ No ○ DK ○ NA *If yes how many Kama ndio, wangapi?* ☐☐

Sheep **(Kondoo)** ○ Yes ○ No ○ DK ○ NA *If yes how many Kama ndio, wangapi?* ☐☐

Goats **(Mbuzi)** ○ Yes ○ No ○ DK ○ NA *If yes how many Kama ndio, wangapi?* ☐☐

Other Animal **(Mnyama mwingine)** ○ Yes ○ No ○ DK ○ NA *If yes how many Kama ndio, wangapi?* ☐☐

7.14 Have any of your family's animals died in the past 30 days?
(do not include animals intentionally slaughtered)

Kuna yoyote kati ya mifugo ya familia yako imekufa katika kipindi cha siku 30 zilizopita?
(usihusishe wanyama waliochinjwa) ○ Yes ○ No ○ DK

If yes, which animals and how many?
Kama ndio, wanyama wepi na wangapi?

		Number adult **Idadi ya wanyama wakubwa**	Number young **Idadi ya wanyama watoto**
Cow **Ng'ombe**	○ Yes ○ No ○ DK	☐☐	☐☐
Goat **Mbuzi**	○ Yes ○ No ○ DK	☐☐	☐☐
Sheep **Kondoo**	○ Yes ○ No ○ DK	☐☐	☐☐
Donkeys **Punda**	○ Yes ○ No ○ DK	☐☐	☐☐
Other specify/ **Wengineo**	○ Yes ○ No ○ DK	☐☐<br>☐☐	☐☐<br>☐☐

If yes to 7.14, proceed to 7.15. If no, skip to SECTION 8
Kama ndio kwa swali 7.14, uliza 7.15. Kama hapana endelea SEHEMU ya 8

7.15 Can you list the causes of death for your animals?
Unaweza kuorodhesha sababu ya vifo kwa wanyama wako?

SECTION 8: WATER USAGE

8.1 What is your primary source of drinking water in the dry & wet seasons?
Chanzo kikuu cha maji ya kunywa kwa wanakaya katika kipindi cha ukame na masika ni kipi?

	Dry season primary source (select one) **Kiangazi, chanzo kikuu (chagua moja tu)**	Dry season, other sources **Msimu wa ukame, vyanzo vingine**	Wet season primary source **Masika, chanzo kikuu (chagua moja tu)**	Wet season, other sources **Msimu wa mvua, vyanzo vingine**
Piped water into the home **Maji ya bomba nyumbani**	O	O	O	O
Piped water near the home **Bomba ndani karibu na boma**	O	O	O	O
Public/ communal well or standpipe **Kisima cha umma, bomba ya umma**	O	O	O	O
River or creek (moving water) directly **Moja kwa moja kutoka katika mto au mfereji (maji yanayotembea)**	O	O	O	O
Lake, pond, dam (standing water) directly **Moja kwa moja kutoka katika ziwa, dimbwi, au bwawa (maji yaliyosimama)**	O	O	O	O
Private well or pump **Kisima au pampu binafsi**	O	O	O	O
From a spring **Moja kwa moja kutoka katika chemchem**	O	O	O	O
Rainwater **Maji ya mvua**	O	O	O	O
Tanker truck **Gari la kubebea maji (boza)**	O	O	O	O
Other (specify) **Vinginevyo (ainisha)**	O	O	O	O

8.2 Is your drinking water treated (by filtering, boiling, chlorinating, straining, etc.)?
Huwa una tibu maji ya kunywa (kwa kuchuja, kuchemsha, kuweka dawa ya klorine, kuchuja kwa nguo, nk)?

O Yes O No

If yes, how frequently do you treat your household drinking water?
Kama ndio, mara ngapi una tibu maji ya kunywa katika kaya yako?

O Always **Kila wakati** O Sometimes **Muda kwa muda** O Never **Hatujafanya chochote** O DK **Sijui**

If always or sometimes, answer 8.3, otherwise skip to SECTION 9
Kama ni "kila wakati" au "muda kwa muda", jibu swali 8.3, vinginevyo nenda SEHEMU ya 9.

8.3 How do you treat it? (choose all that apply)
Kama ndio, unatibu vipi ? (chagua yote yanayohusika)

- O Boiling/ **Kuchemsha**
- O Sedimentation and decant/ **Kuacha kwa muda yatwae/uchafu uende chini**
- O Other, specify/ **Nyinginezo, ainisha**
- O Strain it through a cloth/ **Kuchuja kwa nguo**
- O Solar disinfection/ **Kuweka juani**
- O Adding disinfectant, such as chlorine or bleach **Kuweka dawa kama klorine/ bleach/water guard**
- O Filtering/ **Kuchuja**

SECTION 9: HOME

9.1 What type of toilet system do members of your home normally use? (choose only one)
Ni aina gani ya mfumo wa choo ambao kwa kawaida unatumika na wakazi wa nyumbani kwako? (chagua moja tu)

- ○ Flush or pour toilet with septic tank, including squat toilet **Choo cha kuchuchumaa, cha kumwaga maji na mfumo wa shimo la maji taka**
- ○ Flush or pour toilet connected to sewer pipe **Choo cha maji kilichounganishwa na bomba la maji taka**
- ○ Pit latrine with covering slab **Choo cha shimo kilichosakafiwa**
- ○ Pit latrine without covering slab **Choo cha shimo ambacho hakijasakafiwa (kisichosakafiwa)**
- ○ Ventilated improved pit latrine (VIP) **Choo cha shimo bora chenye bomba la kutoa hewa chafu (VIP)**
- ○ Bucket or plastic bags **Ndoo au mifuko**
- ○ No facilites or field or bush **Hakuna choo wala kwenda porini**

9.2 Do you have electricity in your home?
Una umeme wowote nyumbani kwako? ○ Yes ○ No

If yes, ask question 9.3. If no, skip to 9.4
Kama ndio, uliza swali 9.3. Kama hapana uliza swali 9.4

9.3 What kind of electricity do you have?
Unatumia umeme wa aina gani?

- ○ Grid (Gridi)
- ○ Solar (Solar)
- ○ Generator (Jenerata)
- ○ Other (Mwingineo)

9.4 What type of energy sources are used for cooking in your home? *(primary and secondary sources)*
Aina gani kuu (ya msingi) ya nishati inatumika kwa kupikia nyumbani kwako?

	Primary source (choose one) **Chanzo cha kudumu (Chagua moja)**	Other source (choose all that apply) **Vyanzo vingine (chagua yote yanoyohusika)**
Electricity/ **Umeme**	○	○
Gas/ **Gesi**	○	○
Kerosene/ **Mafuta taa**	○	○
Cow dung/ **Kinyesi cha ng'ombe**	○	○
Firewood/ **Kuni**	○	○
Charcoal/ **Mkaa**	○	○
Other/ **Nyinginezo**	○	○

9.5 Do the members of this home (all combined) own any of the following items? (choose all that apply)
Je, wakazi wa nyumba hii (wote pamoja) wanamiliki chochote kati ya vitu vifuatavyo? (chagua yote yanayohusika)

Please enter 00 in the Number of units field for items that are not owned at this home
Tafadhali jaza 00 katika sehemu ya idadi ya namba kwenye sehemu ambayo vitu hivyo havimilikiwi katika nyumba hii.

	Number of Unit **Ngapi?**	If respondent is Female: **Kama mhojiwa ni mwanamke:** Are any of these items yours personally? **Vitu vyote vyako?**
Ox plough **(Jembe la ng'ombe)**	☐☐	○ Yes ○ No
Ox cart **(Mkokoteni wa ng'ombe)**	☐☐	○ Yes ○ No
Bicycle **(Baiskeli)**	☐☐	○ Yes ○ No
Motorbike **(Pikipiki)**	☐☐	○ Yes ○ No
Car **(Gari)**	☐☐	○ Yes ○ No
Tractor **(Trekta)**	☐☐	○ Yes ○ No
Mobile phone **(Simu ya mkononi)**	☐☐	○ Yes ○ No
Radio **(Redio)**	☐☐	○ Yes ○ No
Television **(Luninga)**	☐☐	○ Yes ○ No
Sofa **(Makochi)**	☐☐	○ Yes ○ No
Bed net **(Chandarua)**	☐☐	○ Yes ○ No
Refrigerator **(Jokofu au friji)**	☐☐	○ Yes ○ No
A Business **(Biashara)**	☐☐	○ Yes ○ No

9.6 How many structures/ buildings including livestock bomas and homes in total are in your boma?
Kuna idadi gani ya mifumo/ majengo, ikiwemo maboma ya mifugo pamoja na idadi ya nyumba kwa ujumla katika boma lako? ☐☐☐

Comments/ **Maelezo:**

Supplementary Figure S1: Details of the *Brucella* detection status, culture bottle type and volume validity for all culture bottles filled for the n=8 individuals from which *Brucella* spp. was isolated from one or more culture bottles. Abbreviations for bottle types as follows: CAS - Castañeda media; SA - Standard Aerobic media; FA - fastidious antimicrobial neutralisation media; FAP - fastidious antimicrobial neutralisation plus media; PF - paediatric fastidious antimicrobial neutralisation; PFP - paediatric fastidious antimicrobial neutralisation plus media; 1 - first inoculated bottle; 2 - second inoculated bottle.

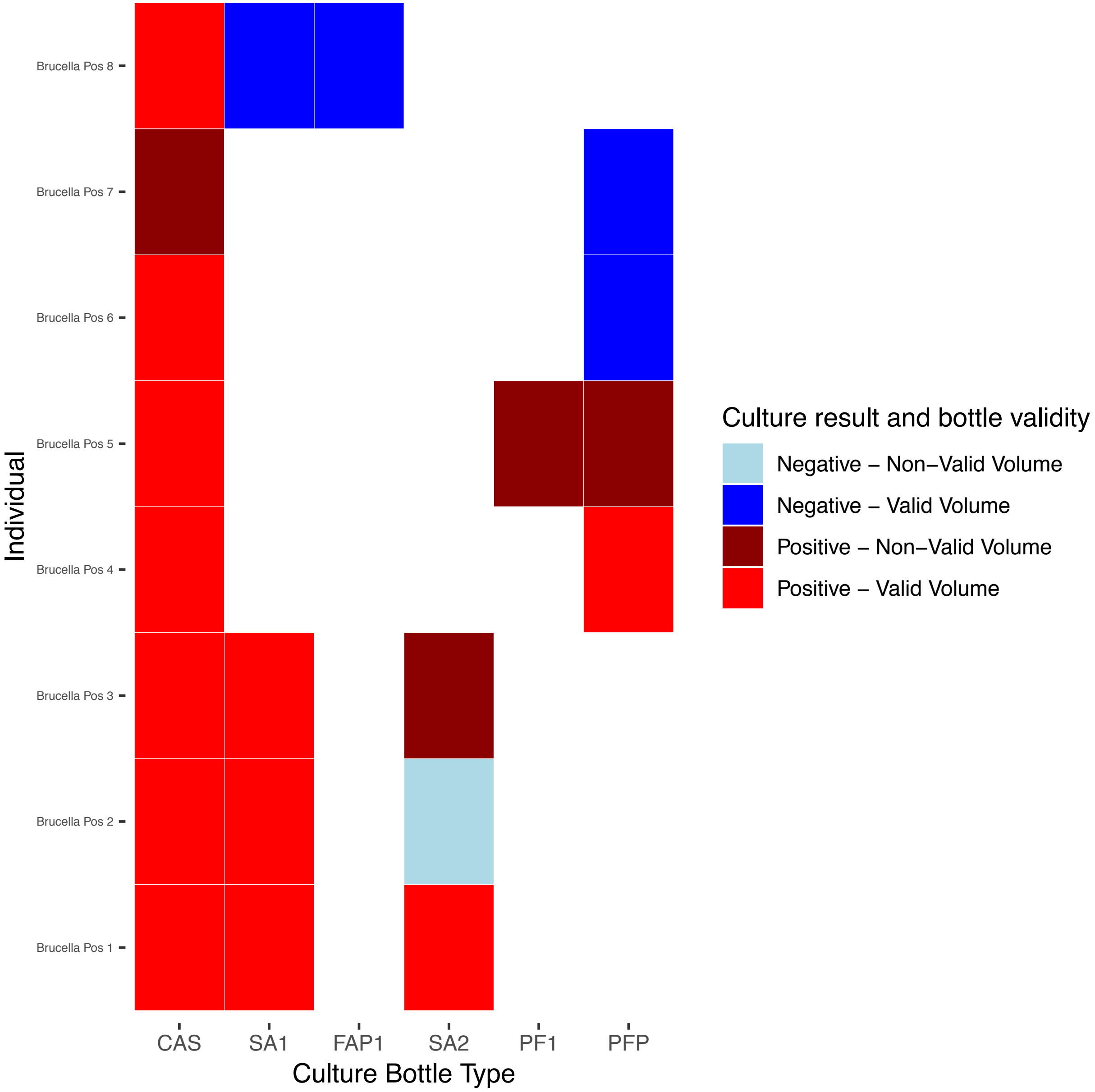

Brucella Pos 8
Brucella Pos 7
Brucella Pos 6
Brucella Pos 5
Brucella Pos 4
Brucella Pos 3
Brucella Pos 2
Brucella Pos 1
Individual
CAS
SA1
FAP1
SA2
PF1
PFP
Culture Bottle Type
Culture result and bottle validity
Negative – Non–Valid Volume
Negative – Valid Volume
Positive – Non–Valid Volume
Positive – Valid Volume

Supplementary Figure S2: Graph showing the log SAT titres for study participants against date of sample collection. Small filled points illustrated in red are acute phase samples and points shown in blue are convalescent phase samples. Lines connecting two points illustrate paired samples collected from the same individual. Solid lines shown in red indicate individuals with rising titres between their acute and convalescent sample. Dashed lines shown in blue indicate individuals with equal or declining titres between their acute to convalescent sample. The black dashed horizontal line indicates the log SAT value equivalent to an SAT titre of 160. Large open circles are plotted around the points for individuals who met criteria for probable brucellosis based on either the acute or convalescent sample (with colour corresponding to the acute or convalescent status of the sample). Points labelled with X indicate the acute sample collection date and acute sample log SAT titre for individuals who were *Brucella* spp. culture positive in samples collected on that date. Brackets towards the top of the plot indicate periods during which all bottles were processed by manual culture methods.

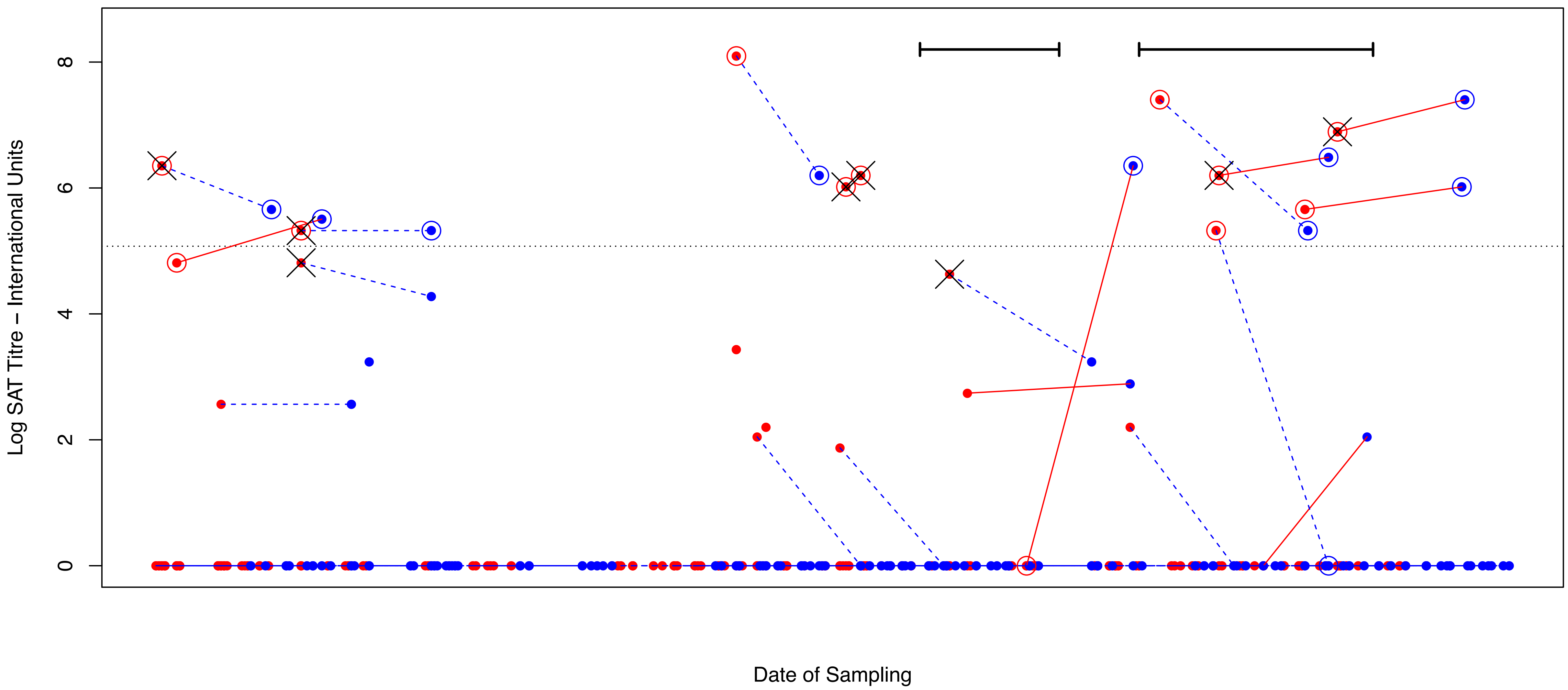
Log SAT Titre – International Units
0
2
4
6
8
Date of Sampling
